# Effects of gravitational loading levels on protein expression related to metabolic and/or morphologic properties of mouse neck muscles

**DOI:** 10.1002/phy2.183

**Published:** 2014-01-13

**Authors:** Tomotaka Ohira, Takashi Ohira, Fuminori Kawano, Tsubasa Shibaguchi, Hirooki Okabe, Katsumasa Goto, Futoshi Ogita, Masamichi Sudoh, Roland Richard Roy, Victor Reggie Edgerton, Ranieri Cancedda, Yoshinobu Ohira

**Affiliations:** 1 Graduate School of Health Sciences, Toyohashi SOZO University, Toyohashi City, 440‐8511, Aichi, Japan; 2 Space Biomedical Research Office, Japan Aerospace Exploration Agency, Tsukuba City, 305‐8505, Ibaraki, Japan; 3 Graduate School of Medicine, Osaka University, Toyonaka City, 560‐0043, Osaka, Japan; 4 Graduate School of Frontier Biosciences, Osaka University, Toyonaka City, Osaka560‐0043, Japan; 5 Faculty of Letters, Kokushikan University, Setagaya‐ku, 154‐0017, Tokyo, Japan; 6 Department of Sports and Life Science, National Institute of Fitness and Sports, Kanoya City, 891‐2393, Kagoshima, Japan; 7 Division of Aerospace Medicine, Department of Cell Physiology, Jikei University School of Medicine, Minato‐ku, 105‐8461, Tokyo, Japan; 8 Department of Integrative Biology and Physiology and Brain Research Institute, University of California, Los Angeles, 90095, California; 9 Universita' degli Studi di Genova & Istituto Nazionale per la Ricerca sul Cancro, Genova City, Italy; 10 Research Center for Adipocyte and Muscle Science, Doshisha University, Kyotanabe City, 610‐0394, Kyoto, Japan

**Keywords:** mouse neck muscle, gravitational unloading, protein expression

## Abstract

The effects of 3 months of spaceflight (SF), hindlimb suspension, or exposure to 2G on the characteristics of neck muscle in mice were studied. Three 8‐week‐old male C57BL/10J wild‐type mice were exposed to microgravity on the International Space Station in mouse drawer system (MDS) project, although only one mouse returned to the Earth alive. Housing of mice in a small MDS cage (11.6 × 9.8‐cm and 8.4‐cm height) and/or in a regular vivarium cage was also performed as the ground controls. Furthermore, ground‐based hindlimb suspension and 2G exposure by using animal centrifuge (*n* = 5 each group) were performed. SF‐related shift of fiber phenotype from type I to II and atrophy of type I fibers were noted. Shift of fiber phenotype was related to downregulation of mitochondrial proteins and upregulation of glycolytic proteins, suggesting a shift from oxidative to glycolytic metabolism. The responses of proteins related to calcium handling, myofibrillar structure, and heat stress were also closely related to the shift of muscular properties toward fast‐twitch type. Surprisingly, responses of proteins to 2G exposure and hindlimb suspension were similar to SF, although the shift of fiber types and atrophy were not statistically significant. These phenomena may be related to the behavior of mice that the relaxed posture without lifting their head up was maintained after about 2 weeks. It was suggested that inhibition of normal muscular activities associated with gravitational unloading causes significant changes in the protein expression related to metabolic and/or morphological properties in mouse neck muscle.

## Abstract (precis)

Inhibition of gravitational loading in space and on the Earth for 3 months caused similar responses of protein expression in mouse neck muscle. Downregulation of mitochondrial proteins and upregulation of glycolytic proteins were induced, suggesting a shift from oxidative to glycolytic metabolism. Furthermore, the responses of proteins, involved in calcium handling, myofibrillar structure, and heat stress, related to the shift of muscular properties toward fast‐twitch type were also noted. It was suggested that inhibition of normal muscular activities associated with gravitational unloading caused significant changes in the protein expression related to metabolic and/or morphological properties in mouse neck muscle.

## Introduction

Removal of weight‐bearing activity induces remarkable effects on the skeletal muscles responsible for maintaining posture and ground support (Roy et al. 1991; Edgerton and Roy 1996; Fitts et al. 2013). For example, chronic unloading of muscles, such as bedrest in humans (Ohira et al. 1999), 2000); Yamashita‐Goto et al. 2001) and hindlimb suspension and actual spaceflight (SF) in animals (Ohira et al. 1992), 2002a),b, 2011); Caiozzo et al. 1994), result in muscle fiber atrophy and a shift toward a faster myosin heavy chain (MHC) profile, particularly in muscles composed predominantly of slow fibers such as the soleus and adductor longus.

These responses appear to be closely related to a decrease in the neuromuscular activity levels, that is, mechanical loading (Riley et al. 1990); Kawano et al. 2004a), 2007) and/or muscle activation (Alford et al. 1987); Kawano et al. 2002), 2004a); Ohira et al. 2002a),b; De‐Doncker et al. 2005) levels. Mitochondrial metabolic properties, which are closely related to the MHC profile, also are related to the neuromuscular activity levels and/or content of high‐energy phosphates in skeletal muscles (Stump et al. 1990); Ohira et al. 1994a),b; Ren et al. 1995); Fitts et al. 2013).

Neck muscles are known to play an important role in maintaining head posture against gravity under 1G conditions (Kawano et al. 2004b). How the properties of neck muscles are influenced by long‐term exposure to microgravity or hypergravity, however, is unknown. We hypothesized that the properties of the neck muscles would be regulated in response to the level of chronic mechanical loading. Therefore, the current study was performed to determine the effects of 3 months of actual SF, hindlimb unloading (a ground‐based model of SF), or 2G conditions on the fiber size and phenotype, and muscle protein expression in the *rhomboideus capitis* of adult mice.

## Methods

### Experimental design and animal care

All experimental procedures were conducted in accordance with the Guide for the Care and Use of Laboratory Animals of the Japanese Physiological Society. The studies were approved by the Committee on Animal Care and Use at Graduate School of Medicine, Osaka University (No. 22‐071) and the Public Veterinary Health Department of the Italian Ministry of Health.

#### SF experiment

Alcatel‐Alenia Space and the Italian Space Agency designed the SF experiment. Three male wild‐type C57BL/10J mice (8 weeks old at launch) were individually housed in mouse drawer system (MDS, 11.6 × 9.8 × 8.4 cm), a payload developed by Alcatel‐Alenia Space (Cancedda et al. 2002). They were launched by space shuttle Discovery (space transportation system No. 128, STS‐128, 28 August 2009) and housed in Japanese Experimental Module (Kibo) on the International Space Station. The mice returned to Kennedy Space Center (KSC) by space shuttle Atlantis (STS‐129, 27 November 2009). Only one mouse returned alive to Earth: the other mice died from an unknown reason on board the International Space Station.

Within 3 h after landing, the surviving mouse was killed by carbon dioxide inhalation and a portion of a neck muscle, *rhomboideus capitis*, was excised bilaterally at the Life Sciences Support Facility at KSC. Excision of the entire muscle was not possible, because the brain and cervical spine were obtained preferentially. Therefore, a comparison of whole muscle weight from the SF mouse with that of other groups was not possible. Responses of hindlimb muscles of the same mice were reported elsewhere (Sandonà et al. 2012).

The muscle from the left side was stretched to a near physiological length and pinned on a cork, frozen in isopentane cooled by liquid nitrogen, and stored on dry ice. Subsequently, a block from the middle of the muscle was mounted on cork using optimum cutting temperature compound, quickly frozen in liquid nitrogen, and stored at −80°C for the determination of fiber cross‐sectional area (CSA) and MHC composition. The muscle from the right side was quick frozen in liquid nitrogen and stored on dry ice for protein expression analysis. The samples were shipped from KSC to Osaka University, Japan, on dry ice.

#### Ground‐based control experiments

Ground‐based experiments were carried out at the Vivarium of the Advanced Biotechnology Center at the University of Genova, Italy, after the completion of flight experiment. To determine any effects of housing in MDS, one wild‐type mouse (ground control, GC) was housed in MDS and another group (*n* = 3) housed in normal vivarium cage (laboratory controls, LC) for 3 months. In addition, 3‐month ground‐based control experiments were performed at Osaka University, Japan. Six groups (*n* = 5/group, except *n* = 4 for the 2G group because one mouse died of unknown cause) of mice were studied: (1) baseline controls (preexperiment), (2) 3‐month hindlimb suspended, (3) 3‐month hindlimb suspended followed by a 3‐month ambulation recovery period, (4) 3‐month exposure to 2G condition in an animal centrifuge, (5) vivarium controls for 3 months, and (6) vivarium controls for 3 months. Sampling and tissue treatment for all mice were performed as done at KSC for the SF mouse.

##### Hindlimb suspension

Ten wild‐type mice (8 weeks old male C57BL/10J) were hindlimb suspended, as described previously (Ohira et al. 2001). Briefly, a narrow piece of tape was secured to the lower third of the tail and a second piece of tape was attached to the tape on the tail that was then connected to a string tied to a horizontal bar at the top of the cage. The string was used to elevate the hindlimbs to prevent any contact with the floor and the walls of the cage during the unloading period. Vivarium control mice were housed in the same animal room for 3 or 6 months (*n* = 5 each). The animal room was maintained at ~23°C and ~55% humidity with 12:12 h light and dark cycle. Food and water were supplied ad libitum.

The hindlimb suspended and vivarium control mice were killed by carbon dioxide inhalation and the *rhomboideus capitis* was excised bilaterally at either 3 or 6 months. The muscles then were prepared for histological and biochemical analyses as described for the muscles from the SF mouse (see above).

##### Exposure to 2G

The mice in the 2G group were loaded continuously for 3 months, except for ∼30 min/day for cleaning and feeding, on a four‐armed animal centrifuge (1.3‐m radius and 38 rpm swing speed) powered by a 0.4‐kW gear motor and controlled by a general‐purpose inverter (Sudoh et al. 1986); Wang et al. 2006). The temperature, humidity, light–dark cycle, and food access conditions were the same as described above. Removal, preparation, and analyses of the *rhomboideus capitis* were the same as described above.

### Fiber phenotype and CSA analyses

Serial cross sections (10‐*μ*m thick) of the muscles from the left side were cut in a cryostat maintained at –20°C and stained immunohistochemically using a mouse on mouse (M.O.M.) basic kit (Vector Laboratories, Burlingame, CA). Muscle sections were fixed with 4% paraformaldehyde for 15 min. Blocking was performed using M.O.M. blocking reagent and 10% donkey serum (Sigma, St. Louis, MO) in 0.1 mol/L phosphate‐buffered saline (PBS) containing 0.1% triton X‐100 for 1 h. The expression of MHC in individual fibers was analyzed using mouse monoclonal antibodies specific to slow (type I) or fast (type II) MHC isoforms, that is, primary antibody NCL‐MHCs and NCL‐MHCf (Novocastra, Newcastle, UK), respectively, as described previously (Ohira et al. 2001), 2011). Laminin, a marker of the basal membrane, was stained using a rabbit antilaminin antibody (Sigma Aldrich, St. Louis, MO).

A fluorescence microscope (BX50) with an argon laser (488 nm peak wavelength) and a He–Ne laser (543 nm peak wavelength) (Olympus, Tokyo, Japan) was used to visualize the stained sections. Serial images of the staining for each antibody were incorporated into a computer and each fiber was classified as type I (slow MHC only), I+II (hybrid of slow and fast MHC), or II (fast MHC only). Approximately 100 fibers were analyzed in both a deep (close to the vertebral column) and superficial (away from the vertebral column) region of each muscle sample.

### Protein expression analyses

Comprehensive analyses of protein expression were performed using the muscles from the right side as reported previously (Santucci et al. 2012). Individual muscle was placed into a mortar and powdered by pestle grinding in liquid nitrogen. The commercial iTRAQ^**®**^ analysis service (Filgen, Nagoya, Japan) was utilized for the mass spectrometric analyses. Briefly, the frozen powder was dissolved in Tissue Protein Extraction Reagent (PIERCE, Rockford, IL). The proteins (100 *μ*g) contained in the extract were digested by trypsin for 24 h at 37°C. The iTRAQ^**®**^ reporter with different molecular weight was conjugated using the iTRAQ^**®**^ reagent‐multiplex assay kit and multiplex buffer kit (AB SCIEX, Framingham, MA) for 2 h at 25°C. The conjugated peptide fragments from each group were combined, separated into eight fractions using the Caution Exchange Buffer Pack (AB SCIEX), and desalted by Sep‐Pak^**®**^ Light C18 Cartridge (Waters, Milford, MA). Subsequently, the mass spectrometric analysis was performed using the QSTAR^**®**^ Elite Hybrid Liquid Chromatography‐tandem Mass Spectrometry (LC/MS/MS) system (AB SCIEX). The peaks obtained in the mass spectrum were analyzed further by the MS/MS measurement. A quantitative comparison was made using the peak level of the reporter molecule observed in the MS/MS spectrum. Database search on the MCBI web site was performed using the obtained mass and sequence of peptides.

### Statistical analyses

#### Body weight, fiber size, and fiber phenotypes

All values were expressed as mean ± SEM. Significant differences were examined by analysis of variance (ANOVA) followed by Scheffé's post hoc test for the body weight, fiber size, and fiber phenotype data obtained at Osaka University. Differences were considered significant at the 0.05 level of confidence. Statistical analysis was not performed for the SF experiment, as the numbers of mice in the LC, GC, and SF groups were 3, 1, and 1, respectively.

#### Protein expression

The peptides, which were matched within the same protein, were pooled and the level in each peak of the reporter was considered as the individual value. Significant differences versus the control group were determined using unpaired *t*‐tests. Differences were considered significant at the 0.1 level of confidence.

## Results

### Body weight

The mean body weights of the cage controls and 2G group increased by 31% and 19%, respectively, during the first 3 months, whereas the mean body weight of the hindlimb unloaded group did not change compared to the preexperimental group (Table 1). After 3 months of ambulatory recovery, the mean body weights of the cage control and hindlimb unloaded groups were 46 and 30% greater than the preexperimental group and 12 and 33% greater than in the C and HS groups, respectively. Data from the SF study are included only for comparative purposes, although it is interesting to note that the one mouse that survived the 3‐month flight had a body weight similar to the age‐matched mice in the LC group.

**Table 1.** Body weight.

3‐month hindlimb suspension and 2G loading experiment (Osaka)					
	R + 0			R + 3 month	
Pre	C	HS	2G	RC	RHS
24.0 ± 0.7 (5)	31.4 ± 0.7^*^ (5)	23.5 ± 0.3 (5)	28.5 ± 0.6^*^ (4)	35.0 ± 0.3^*^^,^^†^ (5)	31.2 ± 1.2^*^^,^^†^ (5)

3‐month spaceflight experiment (Genova)			
	R+0		
Pre	LC	GC	SF
19.1 ± 0.8 (3)	28.5 ± 1.1 (3)	**−**	**−**
19.6 (1)	**−**	24.4 (1)	**−**
22.8 (1)	**−**	**−**	29.3 (1)

Values are mean ± SEM body weight (g). Numbers of mice in each group are shown in parentheses. Pre, preexperimental control; C, cage control; HS, hindlimb suspended; 2G, 2G loaded; RC, age‐matched cage control for RHS; RHS, 3‐month recovery from HS; LC, laboratory control housed in the regular vivarium cage; GC, ground control housed in the mouse drawer system. R + 0 and R + 3 month indicate recovery of 0 and 3 months on the floor.

*and ^†^significantly different from Pre and the respective group at R + 0, respectively, at *P* < 0.05.

### Fiber phenotypes

**Figure 1.**
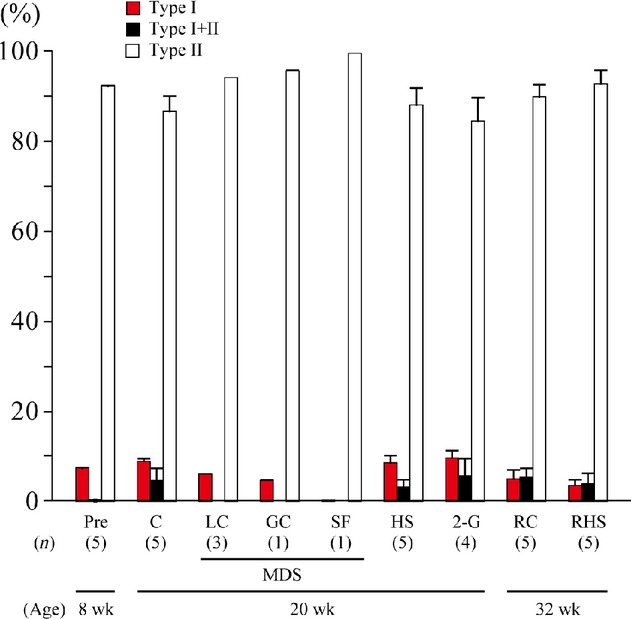
Responses of fiber type distribution in mouse neck muscles. Mean ± SEM. I and II, fibers expressing pure type I and II myosin heavy chain (MHC); I+II, fibers expressing both type I and II MHC; Pre, preexperimental control; C, cage control at the 3rd month in Osaka, Japan; LC, 3‐month laboratory control housed in regular vivarium cage in Genova, Italy; GC, 3‐month control housed in mouse drawer system (MDS) in Genova; SF, spaceflight; HS, hindlimb suspended for 3 months in Osaka; 2G, exposed to 2G for 3 months in Osaka; RHS, recovered from HS on the floor for 3 months; RC, age‐matched cage control housed on the floor for 3 months. (*n*) = number of mice in each group. The age of mice is also shown.

The neck muscles were mainly composed of fast‐twitch fibers expressing only type II MHC (Fig. 1). Although the same gender (male), strain (wild‐type C57BL/10J), and age (8 weeks old at the beginning of experiment, Charles River Laboratories, Wilmington, MA) of mice were utilized for the experiments in Osaka and Genova, fibers coexpressing type I and II MHC were observed only in the mice utilized in Osaka, not in Genova for the MDS project. Therefore, no statistical comparisons were performed between the Genova and Osaka samples.

The fiber type distribution was similar in the LC and GC groups, suggesting that there was no housing effect on the fiber type composition (Fig. 1). Interestingly, the neck muscle in the SF mouse comprised almost exclusively of fibers expressing only the fast MHC isoform. None of the experimental treatments had any significant effect on the fiber type composition in the studies at Osaka University.

### Fiber size

**Figure 2.**
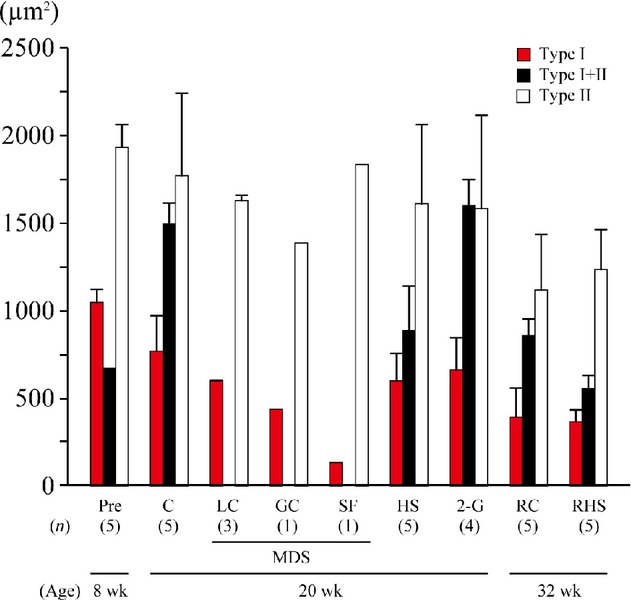
Responses of fiber type–specific cross‐sectional area of neck muscle fibers of mice. Mean ± SEM. See Figure 1 for the abbreviations.

The mean CSA of type I MHC fibers tended to be smaller in the flight mouse (132 *μ*m^2^) than in the LC (595 *μ*m^2^) and GC (441 *μ*m^2^) groups (Fig. 2). The mean fiber CSAs for each fiber type were highly variable and were not significantly affected by hindlimb suspension or 2G exposure.

### Protein expression

**Figure 3.**
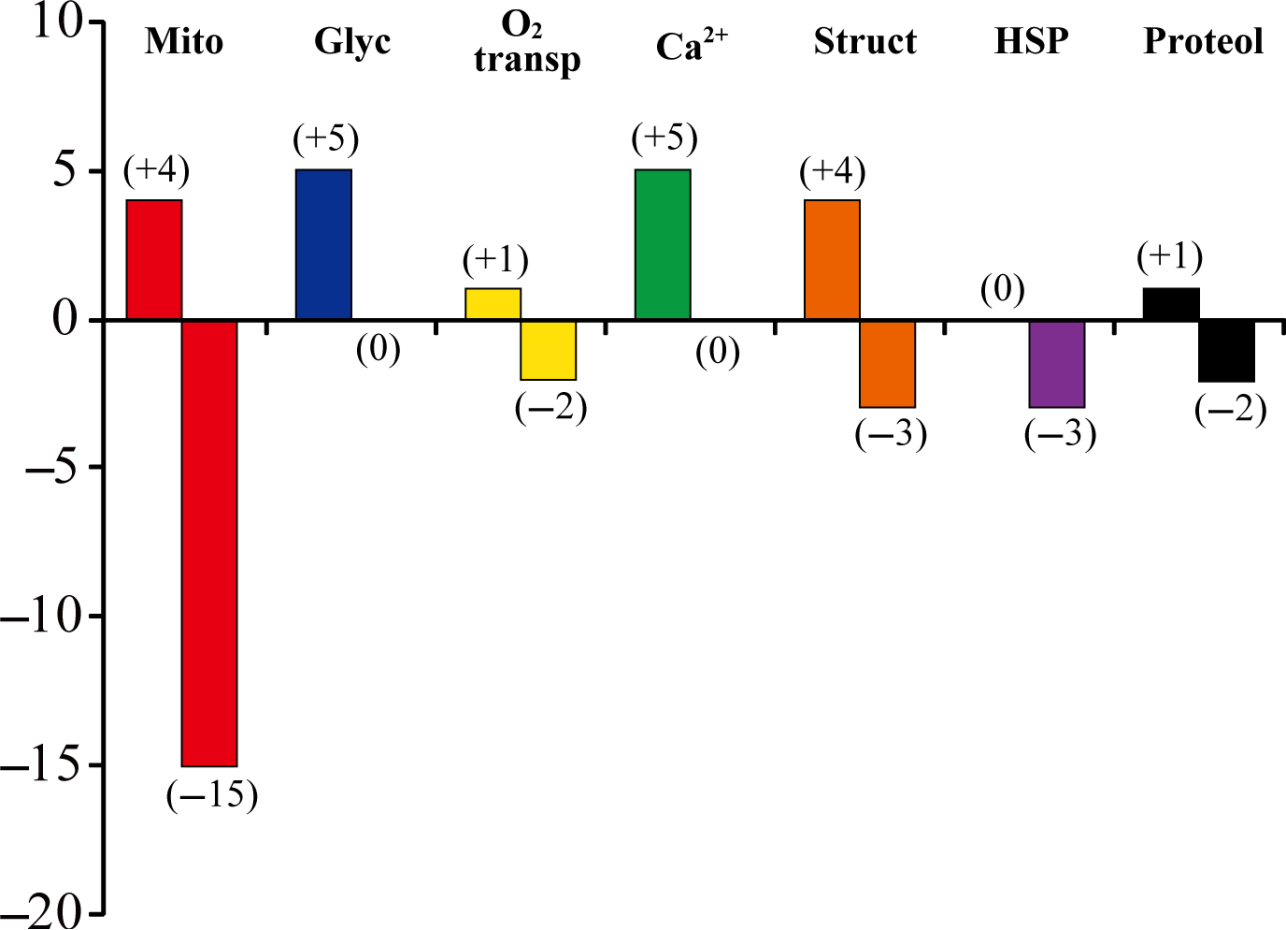
Numbers of up‐ and downregulated proteins in response to spaceflight versus the age‐matched ground‐based vivarium laboratory control. Mito, mitochondria; Glyc, glycolysis; O_2_ transp, oxygen transport; Ca^2+^, calcium metabolism; Struct, myofibrillar structure; HSP, heat‐shock proteins; Proteol, proteolysis.

Protein expression levels in the neck muscles were generally similar in the GC and LC groups (data not shown). The proteins with significant up‐ and downregulation after 3 months of SF, hindlimb suspension, or 2G exposure compared to their age‐matched cage controls are listed in Table 2–Table 8. The numbers of proteins associated with mitochondria, glycolysis, oxygen transport, intracellular calcium handling, myofibrillar structure, heat shock, and proteolysis that were up‐ and downregulated after SF, hindlimb suspension, or 2G exposure are also shown in Figure 3–Figure 5, respectively.

**Table 2.** Changes in mitochondria‐related proteins.

	SF	HS	2G
Upregulation			
ADP/ATP translocase 1	^*^	–	–
ATP synthase subunit b, mitochondrial	^*^	–	–
Cytochrome c oxidase subunit 6B1	–	^*^	^*^
D‐*β*‐hydroxybutyrate dehydrogenase, mitochondrial	–	–	^*^
Dihydrolipoyllysine‐residue acetyltransferase component of pyruvate dehydrogenase complex, mitochondrial	^*^	–	–
NADH dehydrogenase [ubiquinone] 1 *α* subcomplex subunit 9, mitochondrial	–	^*^	–
NADH dehydrogenase [ubiquinone] 1 *α* subcomplex subunit 10, mitochondrial	–	^*^	–
NADH dehydrogenase [ubiquinone] iron‐sulfur protein 2, mitochondrial	–	^*^	–
NADP‐dependent malic enzyme	–	^*^	–
Succinate dehydrogenase [ubiquinone] iron‐sulfur subunit, mitochondrial	^*^	–	–
Downregulation			
Acetyl‐CoA acetyltransferase, mitochondrial	^*^	–	–
Aconitate hydratase, mitochondrial	–	^*^	^*^
Aldehyde dehydrogenase, mitochondrial	–	^*^	^*^
Aspartate aminotransferase, mitochondrial	–	–	^*^
ATP synthase subunit *α*, mitochondrial	–	–	^*^
ATP synthase subunit *β*, mitochondrial	–	^*^	^*^
ATP synthase subunit O, mitochondrial	–	–	^*^
Citrate synthase, mitochondrial	^*^	–	^*^
Coproporphyrinogen‐III oxidase, mitochondrial	^*^	^*^	^*^
Creatine kinase S‐type, mitochondrial	^*^	–	^*^
Delta(3,5)‐delta(2,4)‐dienoyl‐CoA isomerase, mitochondrial	^*^	–	^*^
2,4‐Dienoyl‐CoA reductase, mitochondrial	–	^*^	^*^
Dihydrolipoyl dehydrogenase, mitochondrial	–	–	^*^
Dihydrolipoyllysine‐residue acetyltransferase component of pyruvate dehydrogenase complex, mitochondrial	–	–	^*^
Enoyl‐CoA delta isomerase 1, mitochondrial	^*^	^*^	^*^
Enoyl‐CoA hydratase, mitochondrial	^*^	–	–
10 kDa Heat‐shock protein, mitochondrial	^*^	–	–
Hydroxyacyl‐CoA dehydrogenase, mitochondrial	^*^	^*^	^*^
Isocitrate dehydrogenase [NADP], mitochondrial	^*^	^*^	^*^
Isoform 1 of dihydrolipoyllysine‐residue succinyltransferase component of 2‐oxoglutarate dehydrogenase complex, mitochondrial	–	–	^*^
Isoform 1 of 60 kDa heat‐shock protein, mitochondrial	–	^*^	^*^
Isoform mitochondrial of fumarate hydratase, mitochondrial	^*^	^*^	^*^
Isoform 1 of 2‐oxoglutarate dehydrogenase, mitochondrial	–	^*^	^*^
Long‐chain‐specific acyl‐CoA dehydrogenase, mitochondrial precursor	^*^	–	^*^
Malate dehydrogenase, mitochondrial	^*^	^*^	^*^
Methylmalonate‐semialdehyde dehydrogenase [acylating], mitochondrial	–	–	^*^
Pyruvate dehydrogenase E1 component subunit *α*, somatic form, mitochondrial	–	–	^*^
Pyruvate dehydrogenase E1 component subunit *β*, mitochondrial	–	–	^*^
Pyruvate dehydrogenase phosphatase regulatory subunit, mitochondrial	–	^*^	^*^
28S Ribosomal protein S31, mitochondrial	–	^*^	–
Short‐chain specific acyl‐CoA dehydrogenase, mitochondrial	^*^	^*^	‐
Stress‐70 protein, mitochondrial	^*^	^*^	^*^
Succinyl‐CoA ligase [ADP‐forming] subunit *β*, mitochondrial	–	–	^*^
Superoxide dismutase [Mn], mitochondrial	–	^*^	^*^
Trifunctional enzyme subunit *α*, mitochondrial	–	^*^	^*^
Trifunctional enzyme subunit *β*, mitochondrial	–	^*^	–

Significant responses of protein expressions to spaceflight (SF), hindlimb suspension (HS), or 2G exposure (2G) are indicated by *P < 0.05; −*P* > 0.05.

**Table 3.** Changes in glycolysis‐related proteins.

	SF	HS	2G
Upregulation			
Creatine kinase M‐type	–	^*^	–
Fructose‐bisphosphate aldolase A isoform 1	^*^	^*^	^*^
Glycerol‐3‐phosphate dehydrogenase [NAD+], cytoplasmic	^*^	–	–
l‐lactate dehydrogenase A chain isoform 2	^*^	^*^	–
6‐Phosphofructokinase, muscle type	^*^	–	^*^
Phosphorylase	^*^	–	^*^
Solute carrier family 2, facilitated glucose transporter member 4	–	–	^*^
Downregulation			
****None			

Significant responses of protein expressions to spaceflight (SF), hindlimb suspension (HS), or 2G exposure (2G) are indicated by *P < 0.05; –*P* > 0.05.

**Table 4.** Changes in oxygen transport‐related proteins.

	SF	HS	2G
Upregulation			
Hemoglobin *α*, adult chain 2	–	^*^,^†^	–
Hemoglobin subunit *β*‐1‐like	–	^*^,^†^	–
Myoglobin	^*^	^*^	^*^
Downregulation			
Hemoglobin *α*, adult chain 2	^*^,^†^	–	^*^,^†^
Hemoglobin subunit *β*‐1‐like	^*^,^†^	–	^*^,^†^
Serotransferrin	–	–	^*^

Significant responses of protein expressions to spaceflight (SF), hindlimb suspension (HS), or 2G exposure (2G) are indicated by *P < 0.05; −*P* > 0.05. The ^†^indicates that the response of specific protein was opposite between each group.

**Table 5.** Changes in intracellular Ca^2+^ handling‐related proteins.

	SF	HS	2G
Upregulation			
Calsequestrin‐1	^*^	^*^	–
Isoform 2B of voltage‐dependent calcium channel subunit *α*‐2/*δ*‐1	^*^	–	–
Parvalbumin *α*	^*^	^*^	–
Ryanodine receptor 1, skeletal muscle	^*^	–	–
Sarcoplasmic/endoplasmic reticulum calcium ATPase 1	^*^	^*^	–
Downregulation			
Calsequestrin‐2	–	^*^	–
Ryanodine receptor 1, skeletal muscle	–	–	^*^

Significant responses of protein expressions to spaceflight (SF), hindlimb suspension (HS), or 2G exposure (2G) are indicated by *P < 0.05; −*P* > 0.05.

**Table 6.** Changes in structural proteins.

	SF	HS	2G
Upregulation			
*α*‐actinin‐3	^*^	–	–
*α*‐crystallin B chain	–	–	^*^,^†^
Cofilin‐2	–	^*^	–
Isoform MLC1 of myosin light chain 1/3, skeletal muscle isoform	–	^*^	–
Isoform 1 of titin	–	^*^,^†^	–
Isoform 1 of tropomyosin *α*‐1 chain	^*^,^†^	–	–
Isoform 1 of tropomyosin *β* chain	^*^	–	–
Myomesin 2	–	^*^	–
Myosin‐binding protein C, fast type	^*^	^*^	^*^
Myosin‐binding protein C, slow type	–	–	^*^
Myosin regulatory light chain 2, skeletal muscle isoform	–	^*^	–
Downregulation			
*α*‐actinin‐2	–	^*^	^*^
*α*‐crystallin B chain	^*^,^†^	–	–
Desmin	^*^	–	^*^
Isoform 1 of titin	^*^,^†^	–	^*^,^†^
Isoform 1 of tropomyosin *α*‐1 chain	–	^*^,^†^	–

Significant responses of protein expressions to spaceflight (SF), hindlimb suspension (HS), or 2G exposure (2G) are indicated by *P < 0.05; −*P* > 0.05. The ^†^indicates that the response of specific protein was opposite between each group.

**Table 7.** Changes in heat‐shock proteins.

	SF	HS	2G
Upregulation			
Heat‐shock 70 kDa protein 1A	–	–	^*^,^†^
Heat‐shock protein *β*‐6	–	–	^*^,^†^
Isoform A of heat‐shock protein *β*‐1	–	–	^*^,^†^
Downregulation			
Heat‐shock 70 kDa protein 1A	^*^,^†^	–	–
Heat‐shock 70 kDa protein 4	–	–	^*^
Heat‐shock protein *β*‐6	^*^,^†^	–	–
Isoform A of heat‐shock protein *β*‐1	^*^,^†^	–	–

Significant responses of protein expressions to spaceflight (SF), hindlimb suspension (HS), or 2G exposure (2G) are indicated by *P < 0.05; −*P* > 0.05. The ^†^indicates that the response of specific protein was opposite between each group.

**Table 8.** Changes in proteolysis‐related proteins.

	SF	HS	2G
Upregulation			
Isoform 2 of proteasome‐associated protein ECM29 homolog	^*^	–	–
Downregulation			
*α*‐1‐antitrypsin 1‐5	^*^	–	–
Ubiquitin‐like modifier‐activating enzyme 1	^*^	^*^	–

Significant responses of protein expressions to spaceflight (SF), hindlimb suspension (HS), or 2G exposure (2G) are indicated by *P < 0.05; −*P* > 0.05.

**Figure 4.**
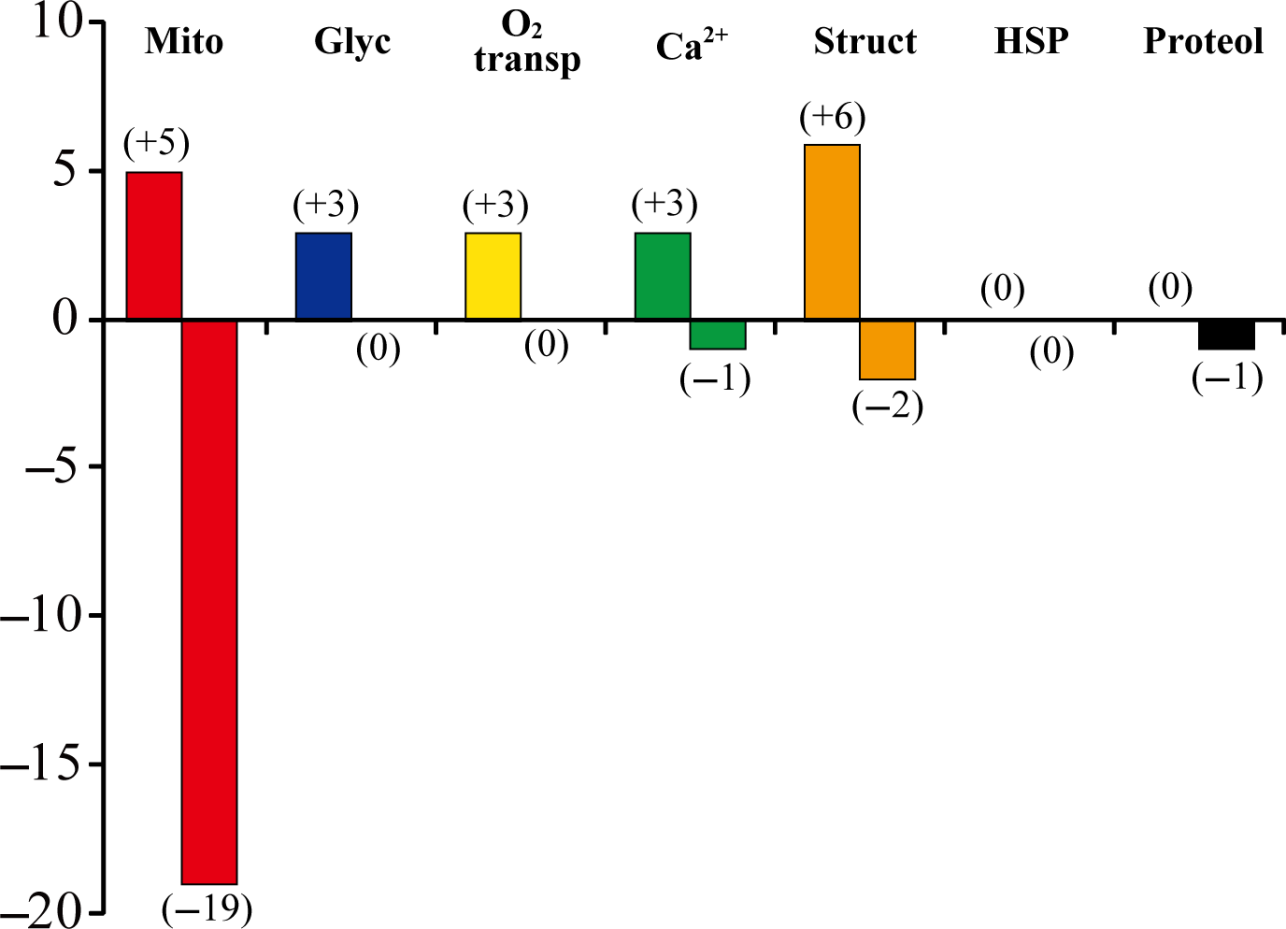
Numbers of up‐ and downregulated proteins in response to hindlimb suspension versus the age‐matched cage control. Mito, mitochondria; Glyc, glycolysis; O_2_ transp, oxygen transport; Ca^2+^, calcium metabolism; Struct, myofibrillar structure; HSP, heat‐shock proteins; Proteol, proteolysis.

**Figure 5.**
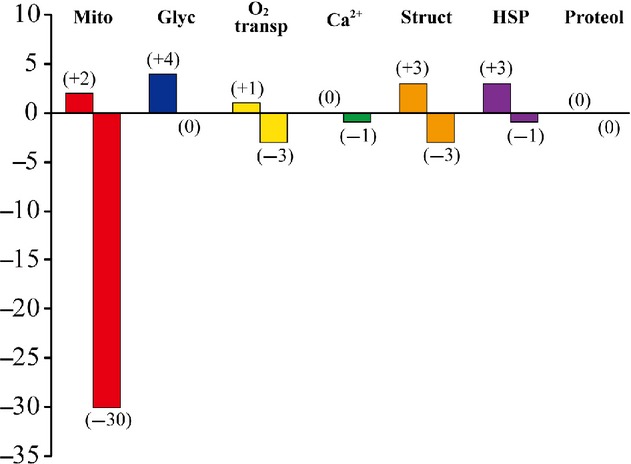
Numbers of up‐ and downregulated proteins in response to 2G exposure versus the age‐matched cage control. Mito, mitochondria; Glyc, glycolysis; O_2_ transp, oxygen transport; Ca^2+^, calcium metabolism; Struct, myofibrillar structure; HSP, heat‐shock proteins; Proteol, proteolysis.

#### Mitochondria‐related proteins

A larger number of mitochondria‐related proteins were downregulated (15–30) versus the upregulated proteins (2–5) across the three conditions (Table 2 and Fig. 3–5). The only coincident response was an upregulation of cytochrome c oxidase subunit 6B1 in the hindlimb suspension and 2G groups (Table 2). Interestingly, different mitochondrial proteins related to ubiquinone were upregulated in the hindlimb‐suspended group.

Several proteins were downregulated in all three or two of the groups. Seven proteins (coproporphyrinogen‐III oxidase, enoyl‐CoA delta isomerase 1, hydroxyacyl‐CoA dehydrogenase, isocitrate dehydrogenase [NADP], isoform mitochondrial of fumarate hydratase, malate dehydrogenase, and stress‐70 protein) were downregulated in all groups. Furthermore, four proteins {citrate synthase, creatine kinase S‐type, delta(3,5)‐delta(2,4)‐dienoyl‐CoA isomerase, and long‐chain‐specific acyl‐CoA dehydrogenase} responded similarly to both SF and 2G exposure. Short‐chain specific acyl‐CoA dehydrogenase was downregulated by both SF and hindlimb suspension. Nine proteins (aconitate hydratase, aldehyde dehydrogenase, ATP synthase subunit *β*, 2,4‐dienoyl‐CoA reductase, isoform 1 of 60 kDa heat‐shock protein, isoform 1 of 2‐oxoglutarate dehydrogenase, pyruvate dehydrogenase phosphatase regulatory subunit, superoxide dismutase [Mn], and trifunctional enzyme subunit *α*) were equally downregulated by hindlimb suspension and 2G exposure.

#### Glycolysis‐related proteins

Similar responses of glycolysis‐related proteins were observed in response to the three different experimental conditions (Table 3 and Figs. 3–5). Five proteins were upregulated by SF {fructose‐bisphosphate aldolase A isoform 1, glycerol‐3‐phosphate dehydrogenase [NAD+] (cytoplasmic), L‐lactate dehydrogenase A chain isoform 2, 6‐phosphofructokinase (muscle type), and phosphorylase} (Table 3). Fructose‐bisphosphate aldolase A isoform 1 was upregulated in all three conditions. L‐lactate dehydrogenase A chain isoform 2 was upregulated by SF and hindlimb suspension. Furthermore, there was an upregulation of 6‐phosphofructokinase (muscle type) and phosphorylase after SF and exposure to 2G. No downregulation of any protein studied was observed under any of the conditions.

#### Other proteins

Upregulation of 1, 5, 4, 0, and 1 proteins, related to oxygen transport, calcium metabolism, myofibrillar structure, heat stress, and proteolysis, was seen in response to SF, respectively (Table 4–Table 8 and Fig. 3). Downregulation was noted in 2, 0, 3, 3, and 2 proteins. The numbers of these proteins, upregulated by hindlimb suspension, were 3, 3, 6, 0, and 0, respectively (Table 4–Table 8 and Fig. 4). Hindlimb suspension caused downregulation of 1, 2, and 1 proteins related to calcium metabolism, myofibrillar structure, and proteolysis, respectively. Furthermore, exposure to 2G caused significant up/downregulation of 1/3, 0/1, 3/3, and 3/1 proteins, related to oxygen transport, calcium metabolism, myofibrillar structure, and heat stress, respectively (Table 4–Table 7 and Fig. 5).

As for the proteins related to oxygen transport, SF, hindlimb suspension, and 2G exposure caused upregulation of myoglobin (Table 4). Hemoglobin *α* (adult chain 2) and hemoglobin subunit *β*‐1‐like were downregulated by both SF and 2G exposure. But these proteins were upregulated by hindlimb suspension, on the contrary.

Three intracellular calcium handling proteins, calsequestrin‐1, parvalbumin *α*, and sarcoplasmic/endoplasmic reticulum calcium ATPase 1, were significantly upregulated by SF and hindlimb suspension, but not by 2G exposure (Table 5). Calsequestrin‐2 was downregulated by hindlimb suspension, on the contrary. Ryanodine receptor 1 (skeletal muscle) was upregulated following SF, but was downregulated by 2G exposure.

As for the structural protein, myosin‐binding protein C (fast type) was equally upregulated by SF, hindlimb suspension, and 2G exposure (Table 6). Downregulation was seen in desmin and isoform 1 of titin following SF and 2G exposure. Furthermore, downregulation, caused by hindlimb suspension and 2G exposure, was noted in *α*‐actinin‐2, although *α*‐actinin‐3 was upregulated by SF. Opposite responses were also seen. Although upregulation of isoform 1 of tropomyosin *α*‐1 chain by SF, isoform 1 of titin by hindlimb suspension, and *α*‐crystallin B chain by 2G exposure was seen, these proteins were, downregulated by hindlimb suspension, SF and 2G exposure, and SF, respectively.

Pronounced responses were not seen in heat‐shock proteins (Table 7). Although heat‐shock 70 kDa protein 1A, heat‐shock protein *β*‐6, and isoform A of heat‐shock protein *β*‐1 were downregulated after SF, they were upregulated by 2G exposure, on the contrary. One of the proteolysis‐related proteins, isoform 2 of proteasome‐associated protein ECM29 homolog was upregulated by SF (Table 8). And SF‐related downregulation of *α*‐1‐antitrypsin 1–5 and ubiquitin‐like modifier‐activating enzyme 1, which was also seen after hindlimb suspension, was observed.

## Discussion

Responses of CSA and MHC phenotypes in individual neck muscle fibers, as well as protein expressions in whole muscle, of male wild‐type C57BL/10J mice to 3‐month SF, hindlimb suspension, or 2G exposure were studied. As the results, it was suggested that inhibition of mechanical muscular activities causes significant changes in the protein expression related to metabolic and/or morphological properties as in slow‐twitch antigravity muscles.

### Morphologic properties

Chronic exposure to microgravity tended to result in atrophy of the slow fibers in the neck muscle, a finding similar to that reported for a number of hindlimb muscles in rodents (Ohira et al. 1992), 2002a), 2011); Caiozzo et al. 1994). The upregulation of isoform 2 of proteasome‐associated protein ECM29 homolog and downregulation of ubiquitin‐like modifier‐activating enzyme 1 and *α*‐1‐antitrypsin 1‐5, which are major protease inhibitors and break down unneeded proteins, are consistent with the atrophic response (Table 8). We also have observed upregulation of genes (ubiquitin C and growth arrest and DNA‐damage‐inducible 45 gamma), which plays one of the major roles in protein degradation and inhibition of growth, in the same muscle after flight (T. Ohira, T. Ohira, F. Kawano, T. Shibaguchi, H. Okabe, K. Goto, F. Ogita, M. Sudoh, R. R. Roy, V. R. Edgerton, R. Cancedda, and Y. Ohira, unpublished observation). Although there was a downregulation of ubiquitin‐like modifier‐activating enzyme 1 after hindlimb unloading and SF, fiber size was unaffected in both the hindlimb‐suspended and 2G groups.

The electromyogram (EMG) activity of the neck muscles in rats was elevated during the first 2–3 weeks of a 9‐week hindlimb suspension (Kawano et al. 2004b) or 2G exposure (T. Ohira, T. Ohira, F. Kawano, T. Shibaguchi, H. Okabe, K. Goto, F. Ogita, M. Sudoh, R. R. Roy, V. R. Edgerton, R. Cancedda, and Y. Ohira, unpublished observation) studies. Subsequently, however, the activity levels were lower than those recorded during normal cage housing. These results were attributed to the observation that at the 2‐week time point and thereafter the mice maintained a calm posture and kept their head on the floor (no lifting of the head), once they were accustomed to the new environment. Although we did not measure EMG activity in the present studies, we speculate that the mice in the hindlimb‐suspended and 2G groups responded similarly and that the activity levels were decreased during SF as reported for other muscles under 0G conditions (Kawano et al. 2004b); Ohira et al. 2009).

### Metabolic properties

The shift toward a faster fiber phenotype after hindlimb suspension generally is associated with a decrease in mitochondrial enzyme activities as measured in whole‐muscle homogenates (Stump et al. 1990); Ohira et al. 1994b); Fitts et al. 2013), reflecting an inhibition of mitochondrial metabolic rate. The integrated activity (fiber CSA × specific activity) of succinate dehydrogenase in individual fibers of the rat adductor longus measured in cross sections was closely related to fiber size, also suggesting that mitochondrial enzyme levels decrease in parallel with fiber atrophy (Ohira et al. 2011).

In this study, several proteins associated with mitochondria were downregulated after SF, hindlimb suspension, or exposure to 2G (Table 2 and Figs. 3–5). These results suggest that mitochondrial energy metabolism might be inhibited as reported previously (Stump et al. 1990); Ohira et al. 1994b); Fitts et al. 2013). Some mitochondrial proteins, however, were upregulated, suggesting a compensatory mechanism. Such compensatory responses have been observed previously. For example, the levels of mitochondrial matrix enzymes were elevated in skeletal muscles of severely iron‐deficient rats that had very low iron‐containing enzyme activities (Ohira et al. 1987). Denjean et al. (1999) also reported an 80% increase in the expression level of mitochondrial uncoupling protein 3 mRNA in the soleus after 2 or 5 weeks of hindlimb suspension. We also observed upregulation, and no downregulation, of glycolytic enzymes under all three conditions (Table 3). The responses of both the mitochondrial and glycolytic proteins are consistent with the trend for a shift toward “faster” fiber phenotypes (Fig. 1).

Interestingly, hemoglobin‐related proteins were downregulated by SF and exposure to 2G and upregulated by hindlimb suspension (Table 4). The upregulation of hemoglobin proteins by hindlimb suspension could be related to the shift of blood toward the head (Ohira et al. 2002a); Morimoto et al. 2013). The downregulation of these proteins by SF may due to a decrease in blood volume (Hargens and Richardson 2009) and is consistent with a reduction in tissue plasminogen activator gene expression (noted in the same muscle in this study, T. Ohira, T. Ohira, F. Kawano, T. Shibaguchi, H. Okabe, K. Goto, F. Ogita, M. Sudoh, R. R. Roy, V. R. Edgerton, R. Cancedda, and Y. Ohira, unpublished data). The upregulation of myoglobin by all three conditions could be a compensatory response to the inhibited oxidative metabolism in the mitochondria.

Three intracellular calcium handling proteins, that is, calsequestrin‐1 (found in fast skeletal muscle), parvalbumin *α*, and sarcoplasmic/endoplasmic reticulum calcium ATPase 1, were significantly upregulated by SF and hindlimb suspension, but not by exposure to 2G (Table 5). Calsequestrin‐2, found in cardiac and slow skeletal muscle, was downregulated by hindlimb suspension and ryanodine receptor 1 (skeletal muscle) was upregulated by SF. These responses, in general, are consistent with a shift toward a faster phenotype.

In general, the levels of heat‐shock protein were upregulated by 2G, downregulated by SF, and unaffected by hindlimb suspension. The downregulation of heat‐shock 70 kDa protein 1A by SF and 2G is consistent with previous reports on models of disuse (Desplanches et al. 2004). Lawler et al. (2006) reported a decrease in HSP70 expression in soleus muscle after 28 days of hindlimb unloading. As stated earlier, the neck muscle activity (EMG) was decreased during 2G exposure and most likely decreased during weightlessness. Importantly, the levels of HSP72 expression are lower in predominantly fast than predominantly slow muscles (Ogata et al. 2003); Desplanches et al. 2004). Thus, these results are consistent with a shift toward a faster phenotype. However, it is not clear why these proteins were upregulated by 2G exposure.

### Other properties

There were a large number of adaptations (both up‐ and downregulation) of structural proteins by all three conditions (Table 6). Myosin‐binding protein C (fast type) was upregulated by all three conditions and *α*‐actinin‐3, which is associated with improved sprint and power performance in athletes (Berman and North 2010), was upregulated by SF. These changes are consistent with a shift toward a faster phenotype.

Downregulation of desmin, isoform 1 of titin, and *α*‐crystallin B chain, localized at Z‐bands of myofibrils, was seen after SF and/or 2G exposure. Powers et al. (2007) reported the loss of myofibrillar proteins in association with fiber atrophy. Decrease in titin (connectin) and *α*‐crystallin B, which is also a small heat‐shock proteins (HSP), in antigravity soleus muscle of rats was caused by hindlimb suspension, which also caused transformation toward fast‐twitch phenotype (Atomi et al. 1991); Goto et al. 2003); Udaka et al. 2007). Responses of these structural proteins may be also related to the shift of muscle toward fast‐twitch type.

The adaptations in the structural proteins may suggest an unloading‐related remodeling of sarcomeres as observed previously in the soleus muscle of hindlimb unloaded rats (Kawano et al. 2004a). When rats are hindlimb suspended, the soleus muscles are passively shortened due to the chronic plantarflexed position of the ankle joints. Under these conditions, the sarcomeres are in a shortened position and tension development of muscle is inhibited (Kawano et al. 2004a). A similar situation may occur in the unloaded neck muscles under each of the three conditions studied. If this were the case, then the large number of adaptations in the structural proteins could be related to this phenomenon.

### Summary and conclusions

Long‐term SF resulted in a shift toward a faster fiber phenotype and atrophy of the slow fibers in the neck muscle of the SF mouse, findings similar to those reported for antigravity muscles, such as soleus (Ohira et al. 1992); Caiozzo et al. 1994) and adductor longus (Ohira et al. 2011). The shift in fiber phenotype was associated with a downregulation of mitochondrial proteins and an upregulation of glycolytic proteins, suggesting a shift from oxidative to glycolytic metabolism. Furthermore, the responses of proteins related to calcium handling, myofibrillar structure, and heat stress were closely related to a shift toward a faster phenotype. Surprisingly, the responses to either the 2G or hindlimb unloading conditions were, in general, similar to those observed after SF. These similarities with SF most likely reflect the behavior of mice, that is, the mice in the hindlimb unloaded and 2G groups maintained a relaxed head posture (no lifting of the head), once they are accustomed to the new environment.

## Conflict of Interest

None declared.

## References

[b1] Alford E. K. Roy R. R. Hodgson J. A. Edgerton V. R. 1987 Electromyography of rat soleus, medial gastrocnemius, and tibialis anterior during hindlimb suspension. Exp. Neurol.; 96:635-649 3582549 10.1016/0014-4886(87)90225-1

[b2] Atomi Y. Yamada S. Strohman R. Nonomura Y. 1991 Alpha B‐crystallin in skeletal muscle: purification and localization. J. Biochem.; 110:812-822 1783614 10.1093/oxfordjournals.jbchem.a123665

[b3] Berman Y. North K. N. 2010 A gene for speed: the emerging role of alpha‐actinin‐3 in muscle metabolism. Physiology (Bethesda); 25:250-259 20699471 10.1152/physiol.00008.2010

[b4] Caiozzo V. J. Baker M. J. Herrick R. E. Tao M. Baldwin K. M. 1994 Effects of spaceflight on skeletal muscle: mechanical properties and myosin isoform content of slow muscle. J. Appl. Physiol.; 76:1764-1773 8045858 10.1152/jappl.1994.76.4.1764

[b5] Cancedda R. Pignataro S. Alberici G. Tenconi C. 2002 Mice Drawer System: phase c/d development and perspective. J. Gravit. Physiol.; 9:P337-P338 15002603

[b6] De‐Doncker L. Kasri M. Picquet F. Falempin M. 2005 Physiologically adaptive changes of the L_5_ afferent neurogram and of the rat soleus EMG activity during 14 days of hindlimb unloading and recovery. J. Exp. Biol.; 208:4585-4592 16326940 10.1242/jeb.01931

[b7] Denjean F. Desplanches D. Lachuer J. Cohen‐Adad F. Mayet M. H. Duchamp C. 1999 Muscle‐specific up‐regulation of rat UCP3 mRNA expression by long‐term hindlimb unloading. Biochem. Biophys. Res. Commun.; 266:518-522 10600534 10.1006/bbrc.1999.1847

[b8] Desplanches D. Ecochard L. Sempore B. Mayet‐Sornay M. H. Favier R. 2004 Skeletal muscle HSP72 response to mechanical unloading: influence of endurance training. Acta Physiol. Scand.; 180:387-394 15030380 10.1111/j.1365-201X.2003.01255.x

[b9] Edgerton V. R. Roy R. R. 1996 721-763In: Fregly M. J. Blatteis C. M. (eds.). Neuromuscular adaptations to actual and simulated spaceflight. Handbook of Physiology. Section 4. Volume 1. Environmental Physiology. Chapter 32 New York, NY Oxford Univ. Press

[b10] Fitts R. H. Colloton P. A. Trappe S. W. Costill D. L. Bain J. L. W. Riley D. A. 2013 Effects of prolonged space flight on human skeletal muscle enzyme and substrate profiles. J. Appl. Physiol.; 115:667-679 23766501 10.1152/japplphysiol.00489.2013

[b11] Goto K. Okuyama R. Honda M. Uchida H. Akema T. Ohira Y. 2003 Profiles of connectin (titin) in atrophied soleus muscle induced by unloading of rats. J. Appl. Physiol.; 94:897-902 12391127 10.1152/japplphysiol.00408.2002

[b12] Hargens A. R. Richardson S. 2009 Cardiovascular adaptations, fluid shifts, and countermeasures related to space flight. Respir. Physiol. Neurobiol.; 169Suppl. 1:S30-S33 19615471 10.1016/j.resp.2009.07.005

[b13] Kawano F. Nomura T. Ishihara A. Nonaka I. Ohira Y. 2002 Afferent input‐associated reduction of muscle activity in microgravity environment. Neuroscience; 114:1133-1138 12379265 10.1016/s0306-4522(02)00304-4

[b14] Kawano F. Ishihara A. Stevens J. L. Wang X. D. Ohshima S. Horisaka M. 2004a Tension‐ and afferent‐input‐associated responses of neuromuscular system of rats to hindlimb unloading and/or tenotomy. Am. J. Physiol. Regul. Integr. Comp. Physiol.; 287:R76-R86 15031139 10.1152/ajpregu.00694.2003

[b15] Kawano F. Wang X. D. Lan Y. B. Yoneshima H. Ishihara A. Igarashi M. 2004b Hindlimb suspension inhibits air‐righting due to altered recruitment of neck and back muscles in rats. Jpn. J. Physiol.; 54:229-242 15541201 10.2170/jjphysiol.54.229

[b16] Kawano F. Matsuoka Y. Oke Y. Higo Y. Terada M. Wang X. D. 2007 Role(s) of nucleoli, phosphorylation of ribosomal protein S6 and/or HSP27 in the regulation of muscle mass. Am. J. Physiol. Cell Physiol.; 293:C35-C44 17182729 10.1152/ajpcell.00297.2006

[b17] Lawler J. M. Song W. Kwak H. B. 2006 Differential response of heat shock proteins to hindlimb unloading and reloading in the soleus. Muscle Nerve; 33:200-207 16258950 10.1002/mus.20454

[b18] Morimoto T., Kawano F., Izawa T., Ohira L., Nishida K., and Ohira Y.. 2013 Intraocular pressure and retinal vascular changes during transient exposure to microgravity in adult rats. The 34th Ann Meet Int'l Gravit Physiol (Abstract, pp. 55–56), Toyohashi, Japan

[b19] Ogata T. Oishi Y. Roy R. R. Ohmori H. 2003 Endogenous expression and developmental changes of HSP72 in rat skeletal muscles. J. Appl. Physiol.; 95:1279-1286 12909603 10.1152/japplphysiol.00353.2003

[b20] Ohira Y. Cartier L. J. Chen M. Holloszy L. O. 1987 Induction of an increase in mitochondrial matrix enzymes in muscle of iron‐deficient rats. Am. J. Physiol.; 253:C639-C644 3479908 10.1152/ajpcell.1987.253.5.C639

[b21] Ohira Y. Jiang B. Roy R. R. Oganov V. Ilyina‐Kakueva E. Marini J. F. 1992 Rat soleus muscle fiber responses to 14 days of spaceflight and hindlimb suspension. J. Appl. Physiol.; 73:51S-57S 1388148 10.1152/jappl.1992.73.2.S51

[b22] Ohira Y. Saito K. Wakatsuki T. Yasui W. Suetsugu T. Nakamura K. 1994a Responses of *β*‐adrenoceptor in rat soleus to phosphorus compound levels and/or unloading. Am. J. Physiol.; 266:C1257-C1262 8203490 10.1152/ajpcell.1994.266.5.C1257

[b23] Ohira Y. Yasui W. Kariya F. Wakatsuki T. Nakamura K. Asakura T. 1994b Metabolic adaptation of skeletal muscles to gravitational unloading. Acta Astronaut.; 33:113-117 11539510 10.1016/0094-5765(94)90115-5

[b24] Ohira Y. Yoshinaga T. Ohara M. Nonaka I. Yoshioka T. Yamashita‐Goto K. 1999 Myonuclear domain and myosin phenotype in human soleus after bed rest with or without loading. J. Appl. Physiol.; 87:1776-1785 10562622 10.1152/jappl.1999.87.5.1776

[b25] Ohira Y. Yoshinaga T. Nonaka I. Ohara M. Yoshioka T. Yamashita‐Goto K. 2000 Histochemical responses of human soleus muscle fibers to long‐term bedrest with or without countermeasures. Jpn. J. Physiol.; 50:41-47 10866696 10.2170/jjphysiol.50.41

[b26] Ohira Y. Tanaka T. Yoshinaga T. Kawano F. Nomura T. Nonaka I. 2001 Ontogenetic, gravity‐dependent development of rat soleus muscle. Am. J. Physiol. Cell Physiol.; 280:C1008-C1016 11245617 10.1152/ajpcell.2001.280.4.C1008

[b27] Ohira M. Hanada H. Kawano F. Ishihara A. Nonaka I. Ohira Y. 2002a Regulation of the properties of rat hind limb muscles following gravitational unloading. Jpn. J. Physiol.; 52:235-245 12230800 10.2170/jjphysiol.52.235

[b28] Ohira Y. Nomura T. Kawano F. Sato Y. Ishihara A. Nonaka I. 2002b Effects of nine weeks of unloading on neuromuscular activities in adult rats. J. Gravit. Physiol.; 9:49-60 14638459

[b29] Ohira T. Terada M. Kawano F. Nakai N. Ochiai T. Gyotoku J. 2009 Neural and/or mechanical responses of adductor longus muscle to exposure to microgravity in Wistar Hannover rats. Jpn. J. Aerosp. Environ. Med.; 46:21-28

[b30] Ohira T. Terada M. Kawano F. Nakai N. Ogura A. Ohira Y. 2011 Region‐specific responses of adductor longus muscle to gravitational load‐dependent activity in Wistar Hannover rats. PLoS ONE; 6:e21044 21731645 10.1371/journal.pone.0021044 PMC3120817

[b31] Powers S. K. Kavazis A. N. McClung J. M. 2007 Oxidative stress and disuse muscle atrophy. J. Appl. Physiol.; 102:2389-2397 17289908 10.1152/japplphysiol.01202.2006

[b32] Ren J.‐M. Ohira Y. Holloszy J. O. Hamalainen N. Traub I. Pette D. 1995 Effects of *β*‐guanidinopropionic acid‐feeding on the patterns of myosin isoforms in rat fast‐twitch muscle. Pflugers Arch.; 430:389-393 7491263 10.1007/BF00373914

[b33] Riley D. A. Ilyina‐Kakueva E. I. Ellis S. Bain J. L. W. Slocum G. R. Sedlak F. R. 1990 Skeletal muscle fiber, nerve, and blood vessel breakdown in space‐flown rats. FASEB J.; 4:84-91 2153085 10.1096/fasebj.4.1.2153085

[b34] Roy R. R. Baldwin K. M. Edgerton V. R. 1991 269-312 *in*In: Holloszy J. (ed.). The plasticity of skeletal muscle: Effects of neuromuscular activity. Exercise and sports sciences reviews 19 Baltimore, MD Williams and Wilkins 1936088

[b35] Sandonà D. G. M. Camerino J.‐F. Desaphy Bianchini E. Ciciliot S. Danieli‐Betto D. 2012 Adaptation of mouse skeletal muscle to long‐term microgravity in the MDS mission. PLoS ONE; 7:e33232 22470446 10.1371/journal.pone.0033232 PMC3314659

[b36] Santucci D. Kawano F. Ohira T. Terada M. Nakai N. Francia N. 2012 Evaluation of gene, protein and neurotrophin expression in the brain of mice exposed to space environment for 91 days. PLoS ONE; 7:e40112 22808101 10.1371/journal.pone.0040112 PMC3392276

[b37] Stump C. S. Overton J. M. Tipton C. M. 1990 Influence of single hindlimb support during stimulated weightlessness in the rat. J. Appl. Physiol.; 68:627-634 2318773 10.1152/jappl.1990.68.2.627

[b38] Sudoh M. Kohno M. Ikawa S. Saiki H. 1986 Changes of cardiopulmonary responses of rats during centrifugal accelerations. Jpn. J. Aerosp. Environ. Med.; 23:59-67

[b39] Udaka J. Ohmori S. Terui T. Ohtsuki I. Ishiwata S. Kurihara S. 2007 Disuse‐induced preferential loss of the giant protein titin depresses muscle performance via abnormal sarcomeric organization. J. Gen. Physiol.; 131:33-41 18166625 10.1085/jgp.200709888 PMC2174161

[b40] Wang X. D. Kawano F. Matsuoka Y. Fukunaga K. Terada M. Sudoh M. 2006 Mechanical load‐dependent regulation of satellite cell and fiber size in rat soleus muscle. Am. J. Physiol. Cell Physiol.; 290:C981-C989 16291821 10.1152/ajpcell.00298.2005

[b41] Yamashita‐Goto K. Okuyama R. Kawasaki K. Fujita K. Yamada T. Nonaka I. 2001 Maximal and submaximal forces of slow fibers in human soleus after bed rest. J. Appl. Physiol.; 91:417-424 11408459 10.1152/jappl.2001.91.1.417

